# Peripheral Repetitive Transcranial Magnetic Stimulation(rTMS) for Idiopathic Facial Nerve Palsy: A Prospective, Randomized Controlled Trial

**DOI:** 10.1155/2022/7536783

**Published:** 2022-07-13

**Authors:** Zicai Liu, Dongling Xie, Xin Wen, Risheng Wang, Quan Yang, Huiyu Liu, Yuchun Shao, Tingting Liu

**Affiliations:** ^1^Department of Rehabilitation Medicine, YueBei People's Hospital, Shaoguan, 512026 Guangdong Province, China; ^2^School of Rehabilitation Medicine GanNan Medical University, Ganzhou, 341000 Jiangxi Province, China

## Abstract

**Purpose:**

The purpose of this study was to evaluate the clinical efficacy of peripheral repetitive transcranial magnetic stimulation (rTMS) in the treatment of idiopathic facial paralysis, to explore an ideal treatment scheme for idiopathic facial paralysis, and to provide evidence for clinical rehabilitation.

**Methods:**

65 patients with idiopathic facial nerve palsy with the first onset were recruited and randomly divided into rTMS group and control group. Both groups received conventional treatment, rTMS group received additional repetitive transcranial magnetic stimulation to the affected side once a day, 5 times a week for 2 weeks. House-Brackmann (HB) grading scale, Sunnybrook facial grading system (SFGS), and modified Portmann scale (MPS) were used to assess facial nerve function before and after treatment, and the time for patients to return to normal facial nerve function and adverse reaction (AR) was also the main observation index.

**Results:**

After a 2-week intervention, HB, SFGS, and MPS increased in both groups (*P* < 0.01); the improvement of HB, SFGS, and MPS in rTMS group was significantly higher than that in control group (*P* < 0.01). The effective improvement rate of the TMS group after 2 weeks was 90.0%, and that of the control group was 53.3%, and the difference was statistically significant (*P* < 0.01).

**Conclusions:**

Repetitive transcranial magnetic stimulation is a safe and effective noninvasive method for the treatment of idiopathic facial paralysis, which can significantly accelerate the recovery of facial nerve function and provide a new treatment idea for further improving the prognosis of patients with idiopathic facial paralysis.

## 1. Introduction

Idiopathic facial nerve paralysis also calls Bell's paralysis (BP) is an acute *idiopathic facial nerve paralysis* of sudden onset [1]. Unilateral facial paralysis is the most common type of peripheral facial paralysis [2]. The annual incidence is about 30 per 100,000 people with facial paralysis [3], and about 29% of patients had sequelae of differing severity [3]; these include symptoms such as facial muscle movements, contracture, cramps, crocodile tears, hearing impairment, or tinnitus [4], and psychosocial, esthetic disturbances [3]; the quality of life of patients was critically affected. Viral infection such as herpes virus or autoimmune disease may be the pathological mechanism of BP [5], and swelling of nerves caused by an inflammatory process may be the primary cause of facial paralysis [6]. Steroids are the most commonly used treatment [7], but there may be contraindications for patients with chronic diseases such as diabetes mellitus (DM), hypertension (HT), and liver and kidney dysfunction [3]. Acupuncture is also one of the most widely used and effective treatments for BP in China [8]. However, acupuncture is invasive and not suitable for patients who are sensitive to pain.

Repetitive transcranial magnetic stimulation (rTMS) is a noninvasive technique that relies on electromagnetic induction to generate electrical currents to achieve the effect of nerve regulation [9], and it has been widely used in the treatment of various neurological and psychiatric disorders, such as depression, chronic pain, migraine, and stroke [10]. rTMS may be a potential treatment for idiopathic facial neuritis, and a study of 51 patients with acute BP showed that transcranial magnetic stimulation had a good effect on the early prognosis of BP patients [11]. Several Chinese studies have similarly indicated the efficacy and safety of rTMS in the treatment of BP, and the impact of rTMS combined with conventional treatment is more prominent than conventional treatment alone [12–14]. Animal studies have shown that noninvasive pulsed magnetic stimulation of the facial nerve can increase cerebral blood flow [15, 16]. Noninvasive magnetic and electrical stimulation can improve the regeneration rate of axons, promote the formation of new synaptic connections, and accelerate the recovery of nerve function after injury [17, 18].

There are few relevant studies on local repetitive transcranial magnetic stimulation in patients with BP, only a few studies have been published in Chinese literature in the past, and the research scheme was not rigorous, and the evidence was not convincing. The purpose of this study was to evaluate the efficacy and safety of rTMS in the treatment of idiopathic facial paralysis and to provide the basis for clinical idiopathic facial neuritis rehabilitation treatment.

## 2. Materials and Methods

### 2.1. Study Design

This study was a prospective, randomized controlled trial. We intend to recruit patients diagnosed with idiopathic facial neuritis or Bell's palsy in the Department of Rehabilitation Medicine and Neurology of YueBei People's Hospital (Shaoguan city, Guangdong Province, China) in 2021, and they were randomly divided into rTMS group and control group. The evaluation and randomization were blind, conducted by three different researchers, so the researchers who evaluated the results were unaware of the randomization and the intervention. The clinical trial was approved by Yuebei People's Hospital Medical Ethics Committee (approval number: KY-2021-075) and this research protocol was also registered in the Chinese Clinical Trial Registry (number: ChiCTR2100052642).

### 2.2. Participants

An outpatient chief (HYL) with 20 years of clinical experience first interviewed and evaluated each patient, followed by electromyography or electroneurogram examination, and images and laboratory tests were conducted on all patients in order to rule out brain lesions and other causes of secondary facial palsy. An investigator (Tingting Liu) then confirms eligibility for inclusion, and eligible BP patients are informed of all study details, and after the informed consent is signed by the patient or his or her legally authorized representative, the patient will be formally enrolled in the clinical trial for relevant intervention, evaluation, and follow-up. The inclusion criteria were as follows: (1) Bell's palsy (idiopathic facial neuritis) was diagnosed and the facial nerve was damaged identified by electromyography; (2) initially onset and unilateral facial paralysis; (3) aged between 18 and 75; (4) onset was within a month, and the grading of House-Brackmann scale was not less than 3 level; and (5) sign informed consent voluntarily. The exclusion criteria were as follows: (1) patients with central facial paralysis; (2) facial nerve paralysis caused by Lyme disease, encephalitis, tumor or trauma and other reasons; (3) delirious and unable to cooperate with the treatment of patients; (4) contraindications of transcranial magnetic stimulation therapy such as pregnancy or intracranial metal foreign body, history of epilepsy, and implantation of pacemaker; and (5) patients with poor treatment compliance. Patients were sequentially numbered, and treatment regiments, and their corresponding numbers were randomly assigned by a computer and placed in opaque envelopes by an investigator not involved in treatment or evaluation.

### 2.3. Interventions

Both groups received routine rehabilitation, including medication (prednisone tablet, 30 mg, once daily, for 5 days; followed by 5 days, the dosage was gradually reduced until discontinuation), antiviral drugs (acyclovir tablets, 0.2 g, 3 times a day, discontinuation after 7 days), neurotrophic agents (mecobalamin tablets, 0.5 mg, 3 times a day, until discharge), and acupuncture at the same points.

**Figure 1:**
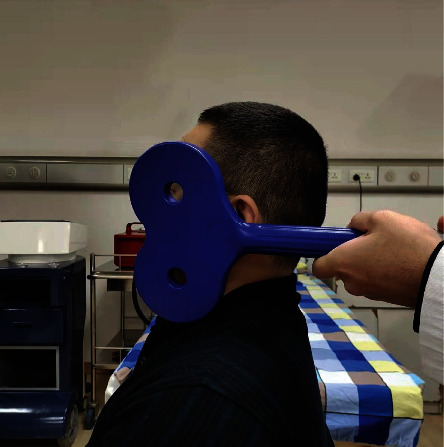
A professional therapist performs rTMS on a patient with facial paralysis, adjusting the position of the treatment coil.

Patients in the rTMS group receive two weeks of rTMS (1 time a day, 5 times a week) on the side of the face with facial paralysis, as shown in Figure 1, and after the therapist positions the coil relative to the patient, the “8” type coil is secured by a metal arm. The coil is positioned on the paralyzed side of the face, between the center of the lower margin of the zygomatic arch and the mandibular notch. Generally speaking, the area covered by the coil exceeds the area of the face or occupies most of the area of the face. When rTMS intervention, the patient's face on the injured side will have obvious stimulation, and the angle and position will be adjusted appropriately if the eyes or teeth are uncomfortable. Our goal is to stimulate the nerves and muscles around the face if the patient can tolerate it. The rTMS group was treated with the NTK-TMS-II transcranial magnetic stimulation instrument (Jiangxi Brain Regulation Technology Development limited-liability company), and the “8” type coil (the coil size:104∗196∗16 mm, Figure 1), 1 time/day, 5 times/week, for a total of 2 weeks (10 times). The parameters of rTMS were as follows: the stimulation frequency of 5 Hz; the stimulation intensity adjusted according to patient tolerance. Patients in the rTMS group received 5-Hz rTMS for 6 s, with an inter-train interval of 14 s, and 60 trains with a total of 1800 pulses

All patients in the rTMS group had resting motor thresholds (RMT) measured before intervention. We use the method of Ilpo Rimpilainen et al. to record motor-evoked potentials (MEP) [19]. The patient is asked to sit quietly and relax; the center of the “8” type coil moves about 3 cm behind the vertex and about 6 cm outside the vertex; disposable electrodes with a diameter of 1 cm were affixed to the nasolabial folds, to record the MEP of TMS; and the coil's hand was always facing posteriorly [11, 19]. RMT is defined as the minimum stimulus intensity of MEP>50 volts in five out of ten consecutive trials [20] of the nasolabial folds. The reference electrodes are located on the same side of the alar nose of the face as the MEP on the injured side may be abnormal. The intensity was adjusted according to the patient's tolerance, so the intensity of stimulation was different for each individual, but the intensity of the stimulation has to be necessary to evoke a motor-evoked potential. We reported the average intensity of stimulation in the rTMS group in Table 1.

### 2.4. Outcome Assessment

Each patient was assessed for facial nerve function by two professionals (Yuchun Shao and Zicai Liu), and the average of the two assessments was taken. The primary outcome measure was Sunnybrook Facial Grading System (SFGS) [21], the secondary outcome measures were House-Brackmann Grading Scale (HBGS) [22–24], and the Modified Portmann Scale (MPS) [25]. In addition, we also recorded the number of adverse events to assess the safety of rTMS for patients with BP.

House-Brackmann grading scale (HBGS) is a scale developed by House and Brackmann for reporting facial function, and it was adopted by the American Society of Otolaryngology-Head and Neck Surgery in 1985. The scale integrates the overall functions of static, dynamic, and linkage, and is relatively simple and widely used in clinical application, which has become the standard for reporting facial nerve results in some otolaryngology journals [24]. HBGS is divided into six levels: level 1 is normal, level 2 is mild, level 3 is moderate, level 4 is moderate to severe, level 5 is severe, and level 6 is complete paralysis. The higher the level, the more severe the facial paralysis [23, 26]. A grade of 1 or 2 HBGS after treatment is considered favorable, while a final HBGS grade ≥3 was considered unfavorable [27, 28].

Sunnybrook facial grading system (SFGS) is a new comprehensive scoring method for facial nerve function proposed by Ross et al. in 1996 [21, 29]. It carefully evaluates facial nerve function from both static and dynamic aspects, and carries out linkage evaluation according to different parts, thus achieving more accurate quantitative scoring. Based on previous studies, SFGS is considered to be the most objective clinical face scoring system currently available [21, 30, 31]. The SFGS table includes three parts: static symmetry, autonomic symmetry, and synkinetic movements. The score ranges from 0 to 100. The higher the score, the better the facial nerve function.

Modified Portmann scale (MPS) includes six autonomous movements (smiling, frowning, eye closing, whistling, shrugging nasal movement, and cheek blowing), with a maximum of 3 points and a minimum of 0 points for each autonomous movement, plus a quiet image score of 2 points for a total of 20 points, and higher scores indicate better facial function [14, 25, 32]. The earliest Chinese literature mentioned this evaluation method in 1996 [33], and it has been widely used by Chinese scholars.

### 2.5. Statistical Analysis and Calculation of Sample Size

The Shapiro-Wilk test was used to determine the normality of the data distribution. Continuous variables were represented by the mean or the median of interquartile spacing; differences between groups at baseline and baseline to the end of the study were examined using the unpaired *t*-test or Wilcoxon signed-ranks test; the intragroup comparison before and after the intervention was performed by paired *t*-test or Wilcoxon-matched pairs signed-ranks test; and for categorical variables, we give counts and percentages. Analysis of variance and *χ*^2^ test were used to compare the baseline data of the included population. The significance level was set as *P* values less than 0.05 on both sides. All analyses were performed using SPSS26.0.

The patient sample size required for this study was calculated through the website (http://hedwig.mgh.harvard.edu/sample_size/size.html) [34]. The previous study [14] has shown that rTMS significantly improved SFGS scores (7.58 ± 3.63) and Portmann scores (1.31 ± 1.75) in BP patients compared with conventional therapy. At a one-sided 0.05 significance level, statistical power is set to 0.8, the sample size was calculated to be at least 46, the probability is 80 percent that the study will detect a treatment difference at a one-sided 0.05 significance level if the true difference between treatments is 1.31 units. This is based on the assumption that the standard deviation of the response variable is 1.75. If 20% of subjects are expected to be lost randomly during the study, a minimum of 58 subjects needs to be recruited.

## 3. Results

**Figure 2:**
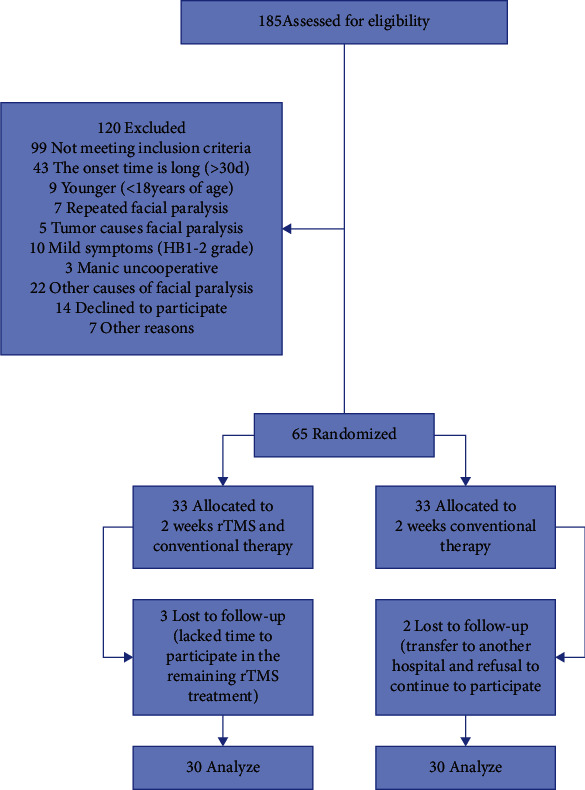
Flow diagram depicting the study design.

Of the 86 patients who met the criteria for enrollment, 65 agreed to participate. 5 of the patients were stopped early. Thus, 60 patients completed all interventions and evaluations and were included in the analysis (Figure 2). There were no statistically significant differences in disease severity, course of disease, left and right facial paralysis, gender, age, height, weight, hypertension, and diabetes between the two groups (*P* > 0.05), and the mean intensity of rTMS group receiving stimulation was 30.83 ± 5.88 (Table 1).

As the treatment progressed, the two groups of MPS scores and SFGS scores were gradually improved, and after treatment, the two groups of patients' facial function assessment of the MPS score and the SFGS score were significantly higher than before treatment (*P* < 0.01); after treatment, the HBGS score was significantly lower than the treatment (*P* < 0.01), indicating that both groups had significantly improved facial functions after two weeks of treatment. The average changes in MPS score (*P* < 0.01, 95% CI [0.688, 4.445]) and SFGS score (*P* = 0.08, 95% CI [10.277, 27.523]) in rTMS groups were significantly higher than that of the control group, and the average variation of HBGS in rTMS group was significantly lower than that of the control group (*Z* = −3.628, *P* < 0.01). (Table 2).

**Figure 3:**
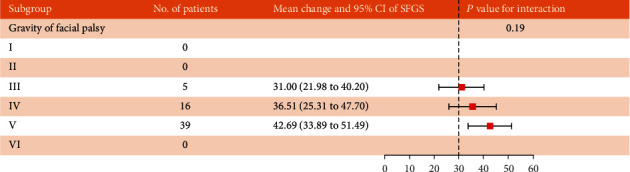
SFGS improvement in patients with different severity of BP.

Figure 3 shows the comparison of SFGS improvement in BP patients with different severity (HBGS). Subgroup analysis showed that there was no significant difference in SFGS improvement among the three HBGS grades (*P* = 0.19).

Table 3 demonstrates the distribution and recovery of HBGS scores in the two groups before and after 14 days of further intervention, and there is no significant difference in the distribution between the two groups before intervention (*χ*^2^ = 3.693, *P* = 0.174). After 14 days, in rTMS group, 7 cases (23.3%) recovered completely within two weeks, in the control group, and 0 cases recovered completely within two weeks. The complete recovery rate was significantly higher in the rTMS group than in the control group (*P* = 0.011). The HBGS grade of rTMS group was significantly better than the control group and was statistically significant (*χ*^2^ = 9.613, *P* < 0.05). If ≥2 grades of HBGS improvement were considered as clinical effective improvement, the effective improvement rate achieved by rTMS was 90.0%, and that achieved by the control group was 53.3%, and the difference was statistically significant (*P* < 0.01).

**Figure 4:**
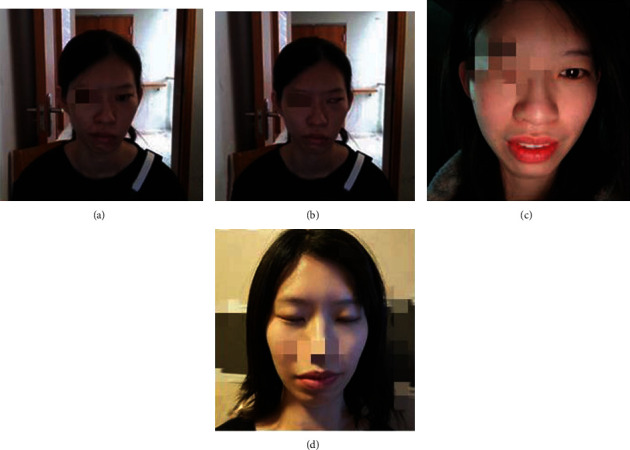
A patient before and after rTMS treatment: (a) and (b) represent the state of open and closed eyes at admission, and (c) and (d) represent the state of follow-up after discharge after rTMS treatment.

Figure 4 shows the recovery of a 32-year-old woman with Bell's palsy. Before therapy, the patient could not close her eyes on the left side, and the corners of her mouth were significantly skewed to the right (a and b). After 2 weeks of rTMS treatment, the patient's eyes were significantly closed and nearly normal (c and d).

All patients who were involved in the study had no serious adverse events, and two cases in rTMS group had a slight toothache, and when appropriately adjusted for the placement of the stimulation coil, the patient was not reported to have a toothache.

## 4. Discussion

Our results suggested that rTMS combined with routine rehabilitation may be better than rehabilitation alone for patients with idiopathic facial neuritis; at the end of the intervention, facial function scores (HBGS, SFGS, and MPS) observed in the rTMS group were significantly better than those in the control group (Table 2). Figure 3 indicates that patients with different severity can get a considerable degree of improvement, and the reason why we speculated that there was no significant difference maybe because at the beginning of the disease, patients with HBGS above grade 3 have the same potential for improvement, and early intervention can achieve large clinical benefits. However, we can still see the trend from the forest map. The higher the level of HBGS, the more room for improvement. After the intervention, the severity distribution and complete improvement rate of HBGS in the rTMS group were better than those in the control group (Table 3).

All patients who completed the treatment does not appear in the process of treatment of epilepsy, headache, dizziness, and other adverse reactions, the two subjects of rTMS group a toothache, but the researchers found that the reason for toothache is to stimulate the coil position is too focused on the teeth; after adjusting the position, no other discomfort, not because of side effects people who dropped out of the test, these results indicate that rTMS has good safety and high compliance in the treatment of idiopathic facial paralysis. Unlike conventional rTMS applications, this was a peripheral facial transcranial magnetic stimulation.

Compared with previous studies, our approach is more rigorous and scientific. To our knowledge, this is the first clinical randomized controlled trial of peripheral facial rTMS in which a new peripheral stimulation target was used and reasonable sample size was calculated. Current treatments for facial paralysis include pharmacological therapy, physiotherapy for facial nerve-muscle retraining, and surgical interventions for facial restoration through dynamic and static techniques [35]. Medications include hormone therapy and neurotrophic medications as well as antiviral medications [36]. Medium-frequency electrical stimulation and ultrashort wave therapy [37], acupuncture [38], facial muscle training, and mirror therapy [39, 40] have also been reported as effective treatments [41]. Overall, early treatment remains limited, and reports of recovery are mixed. The rTMS, a new technology developed in 1992, can have functional effects on multiple parts such as the brain's local and distant cortex, and even reconstruct brain cortical function, and the biological effects generated after stimulation cessation can continue for some time [10, 42].

As for the mechanism of rTMS in the treatment of Bel's palsy, we speculated that the facial damaged nerve could improve the local blood circulation to some extent under the stimulation of central and peripheral magnetic field, and the facial nutrients were supplied again, which could promote the recovery of nerve function, thus achieving the effect of alleviating symptoms and restoring facial nerve function [43]. Manli Sun reported for the first time in the conference that TMS was used to treat idiopathic facial neuritis in children. It was found that rTMS was safe and effective [44]. We stimulated the same site, the anterior part of the affected lateral ear, and found that it was also safe and effective in adults. In recent years, more and more scholars in China have studied the application of rTMS in patients with BP. The area of stimulation was the facial area in front of the ear on the affected side, or the facial motor area in the anterior central gyrus of the brain [14], or the mastoid process at the lateral nerve outlet [45]. The efficacy of rTMS stimulation at different sites in patients with Bell's palsy is unknown.

This study proved the efficacy and safety of rTMS in the treatment of BP and expanded the clinical application of rTMS. However, our study has the following limitations: First of all, HBGS, SFGS, and MPS are used for outcome indicators. If there are electrophysiological indicators, the evaluation results will be objective. Secondly, the HB grade of the selected patients was at least grade 4 or above within 1 month of onset, which may lead to differences in the efficacy of patients with a different course of the disease. Future studies need to further subdivide the course of disease. In addition, the intensity of TMS varies from person to person, which may lead to a certain bias. After all, there are many facial nerves, and they are very sensitive, and the intensity of each person's acceptance is not consistent, so it is difficult to uniform the intensity. In short, repetitive transcranial magnetic stimulation seems to have a good positive impact in improving facial paralysis and need more high-quality randomized controlled study to further expand its application. It is unclear whether the effect of transcranial magnetic stimulation parameters of different interventions, for different ages, and the curative effect of sequelae whether there are differences also need further research.

## 5. Conclusions

rTMS may be a safe and effective noninvasive method for the treatment of idiopathic facial paralysis, which can significantly accelerate the recovery of facial nerve function and provide a new treatment idea for further improving the prognosis of patients with idiopathic facial paralysis.

## Figures and Tables

**Table 1:** Baseline demographics and clinical characteristics.

Characteristic	Mean (SD) or median (IQR)		*P* value
	rTMS group (*n* = 30)	Control group (*n* = 30)	
Age (years)	45.87 (11.68)	51.30 (15.55)	0.131
Sex, male (%)	12 (40.0%)	17 (56.7%)	0.196
Facial paralysis side, left (%)	15 (50.0%)	13 (43.3%)	0.605
Course of disease (day)	5.50 (2.00, 11.25)	4.00 (2.00, 8.00)	0.177
Height (cm)	160.00 (156.75, 165.50)	165.00 (160.00, 168.50)	0.148
Weight (kg)	60.00 (54.38, 70.00)	62.50 (58.50, 73.25)	0.455
Hypertension (%)	7 (23.3%)	7 (23.3%)	1.000
Diabetes (%)	5 (16.7%)	4 (13.3%)	1.000
HBGS	5.00 (4.75, 5.00)	5.00 (4.00, 5.00)	0.074
SFGS	20 (13.75, 27.25)	25.5 (15.50, 47.00)	0.211
MPS	5.00 (4.00, 6.00)	5.00 (3.75, 7.25)	0.719
Average intensity (%)	30.83 (5.88)	/	/

HBGS: House-Brackmann grading scale; SFGS: Sunnybrook facial grading system; MPS: modified Portmann scale; IQR: interquartile range; SD: standard deviation.

**Table 2:** Assessment of facial function from baseline to end of the intervention and the amount of change.

	rTMS group (*n* = 30) Mean (SD)			Control group (*n* = 30) Mean (SD)			*P* value (rTMS VS control)
	Pretreatment	Posttreatment	Change	Pretreatment	Posttreatment	Change	
HBGS	*4.70 (0.60)*	*2.23 (0.90)*∗	*2.47 (0.90)*	*4.43 (0.68)*	*2.83 (0.75)*∗	*1.60 (0.72)*	*P* < 0.01
SFGS	*23.20 (13.35)*	*75.33 (21.66)*∗	*52.13 (19.50)*	*30.77 (17.94)*	*64.00 (23.15)*∗	*33.23 (13.15)*	*P* < 0.01
MPS	*5.33 (2.25)*	*14.93 (4.11)*∗	*9.60 (4.06)*	*5.73 (2.91)*	*12.77 (4.20)*∗	*7.03 (3.13)*	*0.008*

∗Represents comparison between before and after treatment in the same group, *P* < 0.01.

**Table 3:** Comparison of HBGS grade and recovery in the two groups/*n* (%).

	HBGS-I	HBGS-II	HBGS-III	HBGS-IV	HBGS-V
Pretreatment					
rTMS group (30)	0	0	2 (6.7%)	5 (16.7%)	23 (76.7%)
Control group (30)	0	0	3 (10.0%)	11 (36.7%)	16 (53.3%)
*χ* ^2^	3.693				
*P*	0.174				

Posttreatment					
rTMS group (30)	7 (23.3%)	11 (36.7%)	10 (33.3%)	2 (6.7%)	0
Control group (30)	0	11 (36.7%)	13 (43.3%)	6 (20.0%)	0
*χ* ^2^	9.613				
*P*	0.020				

## Data Availability

The full data set generated or analyzed in this study can be obtained by contacting Tingting Liu or Huiyu Liu, the corresponding authors.
